# An Evaluation of the Spontaneous Proliferation of Peripheral Blood Mononuclear Cells in HTLV-1-Infected Individuals Using Flow Cytometry

**DOI:** 10.5402/2011/326719

**Published:** 2011-12-05

**Authors:** Lorena Ana Pinto, Bernardo Galvão Castro, Milena Botelho Pereira Soares, Maria Fernanda Rios Grassi

**Affiliations:** ^1^Advanced Laboratory of Public Health, Gonçalo Moniz Center, Oswaldo Cruz Foundation, 40296-710 Salvador, BA, Brazil; ^2^HTLV Center, Bahiana School of Medicine and Public Health (EBMSP), 40290-000 Salvador, BA, Brazil; ^3^Laboratory of Tissue Engineering and Immunopharmacology, Gonçalo Moniz Center, Oswaldo Cruz Foundation, 40296-710 Salvador, BA, Brazil; ^4^Center of Biotechnology and Cell Therapy, São Rafael Hospital, 41250-390 Salvador, BA, Brazil

## Abstract

The spontaneous proliferation of peripheral blood mononuclear cells (PBMCs) is a hallmark of the human T-lymphotropic virus (HTLV) type-1. Cell proliferation is usually measured using a [^3^H]thymidine incorporation assay. This study aims to quantify the spontaneous proliferation of PBMCs using flow cytometry. PBMCs were cultured for 24 to 120 hours in the presence of 5,6-carboxyfluorescein diacetate succinimidyl ester (CFSE). For comparison, PBMCs were also cultured with [^3^H]thymidine. The cutoff values for spontaneous proliferation were >0.06 for the division index and >5.8% for the percentage of divided cells. Sixty-two percent of HTLV-1-infected individuals presented spontaneous proliferation of PBMCs, which was detected in the first 24 hours. Moreover, proliferation was detected in CD4^+^ and CD8^+^ T-lymphocyte subsets. A positive correlation was found between the division index and [^3^H]thymidine incorporation. This method may prove useful to better understand the phenomenon of spontaneous proliferation of PBMC of patients infected with HTLV-1.

## 1. Introduction

Human T-lymphotropic virus type 1 (HTLV-1) is the etiologic agent of adult T-cell leukemia lymphoma (ATLL) and HTLV-1-associated myelopathy/tropical spastic paraparesis (HAM/TSP) and is associated with uveitis and infective dermatitis in children [1–5]. One of the immunological hallmarks of HTLV infection is the *in vitro* spontaneous proliferation of peripheral blood mononuclear cells (PBMCs), as well as a high production of cytokines such as IFN-*γ*, TNF-*α*, IL-10, and IL-2 [6–11]. HTLV-1 preferentially infects CD4^+^ T lymphocytes, and the spontaneous proliferation of these cells sustains viral load, since newly infected cells are mainly derived by the clonal expansion of infected lymphocytes [7]. There is evidence that both the intensity of spontaneous proliferation and the proviral load are higher in HAM/TSP patients when compared to asymptomatic carriers [12–16]. 

Classically, the intensity of cell proliferation is measured using a [^3^H]thymidine incorporation assay, which requires the handling of carcinogenic and radioactive material. A proliferation assay based on cell staining with 5,6-carboxyfluorescein succinimidyl ester (CFSE) and flow cytometry analysis has been described as an alternative approach [7, 17]. This method has been employed in a wide range of applications, including the detection of T-cell responses against autoantigens and the activity of regulatory T cells [18, 19]. However, the applicability of this assay in HTLV-1-infected patients has not been established. This study aims to quantify the spontaneous proliferation of PBMCs in HTLV-1-infected individuals using flow cytometry as a means to facilitate the understanding of the mechanism that trigger this phenomenon in HTLV-1-infected individuals.

## 2. Materials and Methods

### 2.1. Patients

Forty-five HTLV-1-infected individuals from HTLV-1 Reference Center of Science Development Foundation (Salvador, BA, Brazil) were included in the study. Serum samples were screened for the presence of HTLV-1/2 antibodies by using an enzyme-linked immunosorbent assay (ELISA) (Ab-Capture ELISA test system; Ortho-Clinical Diagnostics, Inc., Raritan, NJ) and confirmed by Western blot assay (HTLV Blot 2.4; Genelabs Technologies, Singapore). Blood samples from 14 healthy individuals from the Gonçalo Moniz Research Center were used as controls. Informed consent was obtained from all enrolled patients, and the institutional research board of the Oswaldo Cruz Foundation (FIOCRUZ) approved this study.

### 2.2. Media and Reagents

RPMI 1640 medium (Sigma Chemical Co., St. Louis, Mo) was supplemented with 2 mM L-glutamine (SIGMA), 1% nonessential amino acids (GIBCO), 1 mM sodium pyruvate (SIGMA), 100 U/mL penicillin (SIGMA), 100 *μ*g/mL streptomycin (SIGMA), 100 *μ*g/mL HEPES (Invitrogen), and fetal calf serum (10%; Hyclone, Logan, UT).

### 2.3. Cells

Peripheral blood mononuclear cells (PBMCs) from individuals were obtained from heparinized venous blood samples by density gradient centrifugation using SepCell (LGC Biotechnology, São Paulo, SP, Brazil). All experiments were performed with freshly isolated PBMC.

### 2.4. Cell Proliferation Assay

The PBMC of HTLV-1-infected individuals were cultured in RPMI 1640 culture medium with 10% AB serum. The PBMC, from healthy individuals were cultured in RPMI 1640 culture medium in the presence of 10% AB serum and 1 U/mL of IL-2 (Sigma Chemical Co., St. Louis, Mo, USA). Cells were cultured using 96-well U-bottom culture plates (Costar, Cambridge, mass) in triplicate at 37°C in a 5% CO_2_ humidified atmosphere for 5 days. The culture medium was changed on the third day. To evaluate cell proliferation, PBMC, were labeled before culture with 5,6-carboxyfluorescein diacetate succinimidyl ester (CFSE) (5 *μ*M) (Invitrogen, Eugene, USA) according to the manufacturer's instructions and maintained in culture during 24, 48, 72, 96, and 120 hours. Then, cells were harvested and washed twice in 2 mL of phosphate-buffered saline containing 1% bovine serum albumin. After a final wash, cells were fixed in phosphate-buffered saline containing 4% paraformaldehyde. To evaluate the proliferation of T-lymphocyte subsets, PBMCs were stained with monoclonal anti-CD3^PE-Cy5^ (BD Biosciences), anti-CD4^PE^ (Caltag), and anti-CD8^APC^ (BD Biosciences) antibodies. Then, cells were fixed in phosphate-buffered saline containing 4% paraformaldehyde. Analyses were performed using a FACSAria (Becton Dickinson, Mountain View, Calif, Ohio, USA) and FlowJo software (Ashland, USA). At least 10^5^ events were analyzed per sample. Proliferation intensity was determined through the percentage of divided cells and the cell division index (Figure 3). The percentage of divided cells was considered the percentage of the cells of the original sample which divided (assuming that no cells died during the culture), while division index was the average number of divisions that a cell (that was present in the starting population) has undergone.

Additionally, cells from 11 HTLV-1-infected individuals were cultured for 5 days and pulsed overnight with 1 *μ*Ci [^3^H]thymidine (specific activity, 2 Ci/mmol; ICN, Costa Mesa, Calif, USA). After this period, the content of the plate was harvested to determine the [^3^H]thymidine incorporation using a Beta Radiation Counter (*β*-matrix 9600, Packard, Meriden, Conn, USA). Results were expressed as mean counts per minute.

### 2.5. Statistical Analyses

Data were expressed as percentages, means, standard deviations, medians, and 25% and 75% percentiles. Groups were compared using the Mann-Whitney test and Kruskal-Wallis test. Correlations were evaluated by Spearman correlation. A *P* value of less than 0.05 denoted a statistically significant difference. Graphad Prism 5.0 software was used for all statistical analyses.

## 3. Results

**Figure 1:**
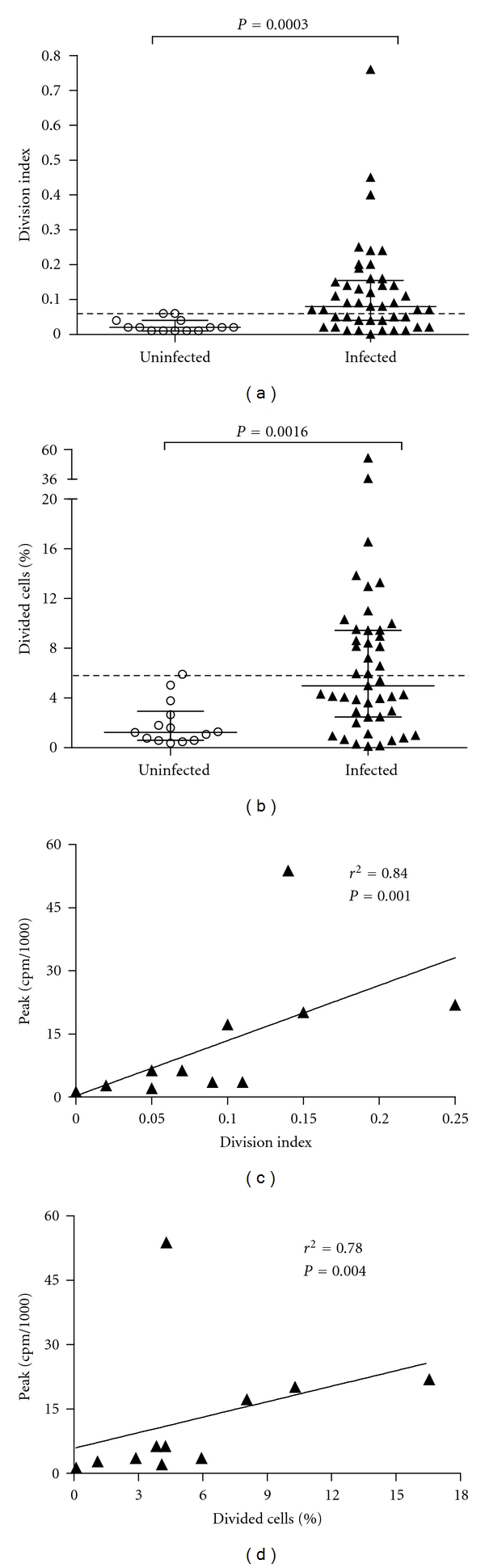
Evaluation of spontaneous proliferation of PBMC from HTLV-1-infected individuals using flow cytometry. (a) Division index of cells. (b) Percentage of divided cells. Uninfected (*n* = 14) and HTLV-1-infected individuals (*n* = 45). The cutoff values to spontaneous proliferation were >0.06 for index division of cells and >5.8% for percentage of divided cells (three times the mean of index division of cells and percentage of divided cells for uninfected PBMC). (c) Correlation between the index division of cells and [^3^H]thymidine incorporation (peak cpm/1000). (d) Correlation between the percentage of divided cells and [^3^H]thymidine incorporation (peak cpm/1000). Differences were considered significant when *P *was <0.05 (Mann-Whitney *U* test). Spearman's correlation coefficients were used.

The cutoff values to define spontaneous proliferation were >0.06 for the cell division index and >5.8% for the percentage of divided cells, which represents three times the division index mean and three times the percentage of divided cells in PBMCs from uninfected controls. Under these criteria, 62% of HTLV-1-infected individuals presented spontaneous proliferation in PBMCs as measured by the cell division index, while 47% presented proliferation using the percentage of divided cells (Figure 1).

Cell proliferation was detected in the first 24 h and remained constant over 120 h (*P* = 0.9) (Table 1).

**Figure 2:**
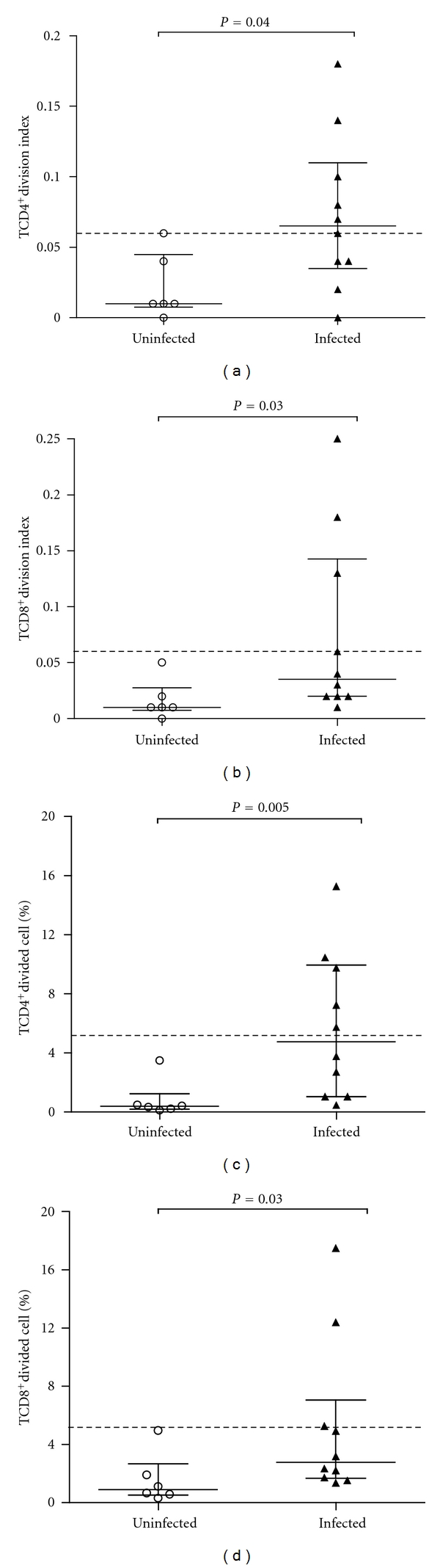
Evaluation of spontaneous proliferation of lymphocyte subsets from HTLV-1-infected individuals using flow cytometry. (a) Division index of CD4^+^ T-lymphocytes subset. (b) Division index of CD8^+^ T-lymphocytes subset. (c) Percentage of divided CD4^+^ T-lymphocytes subset. (d) Percentage of divided CD8^+^ T-lymphocytes subset. Uninfected (*n* = 06) and HTLV-1-infected individuals (*n* = 10). The cutoff values to spontaneous proliferation of CD4^+^ and CD8^+^ T-lymphocytes subsets were >0.06 for index division of cells and >5.2% for percentage of divided cells (three times the mean of index division and % of divided cells for uninfected PBMC).

**Figure 3:**
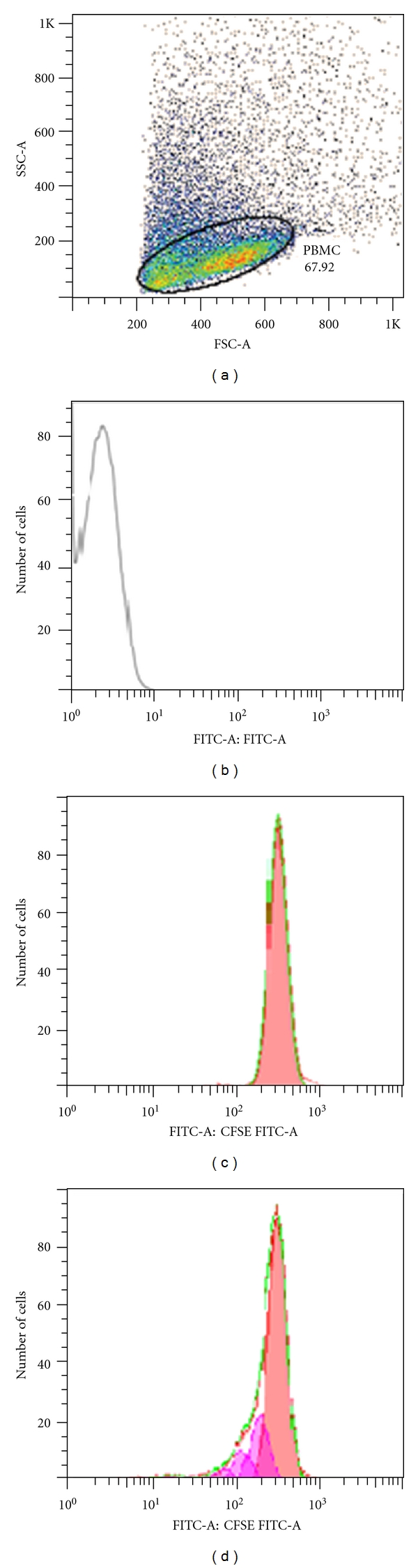
Flow cytometry profiles for evaluation of spontaneous proliferation of PBMCs by CFSE and analyzed by FlowJo software. (a) PBMCs selected for analysis. (b) Unstained cells. (c) An example of curves generated by CFSE-stained PBMC representing cells does not proliferate. (d) An example of curves generated by CFSE-stained PBMC represents proliferation. Each peak represents a cycle cell division. The curves generated by the CFSE profile were analyzed using the proliferation platform of the FlowJo software.

Additionally, using flow cytometry, the proliferation of both CD4 and CD8 T-lymphocyte subsets could be quantified. The cutoff values to define spontaneous proliferation in CD4^+^ and CD8^+^ T-lymphocyte subsets were similar to those defined in the PBMC quantification: >0.06 for the cell division index of cells and >5.2% for the percentage of divided cells. Under these criteria, 50% of HTLV-1-infected individuals presented spontaneous proliferation of CD4^+^ T lymphocytes, while 30% presented spontaneous proliferation of CD8^+^ T lymphocytes (Figure 2).

A positive correlation was found between the division index of cells and [^3^H]thymidine incorporation (*r* = 0.84; *P* = 0.001), as well as between the percentage of divided cells and [^3^H]thymidine incorporation (*r* = 0.78; *P* = 0.004) (Figure 1).

## 4. Discussion

This study determined the parameters to quantify the cell proliferation of PBMC from patients infected with HTLV-1 by flow cytometry. The results obtained indicated that there was a strong correlation between the intensity of spontaneous proliferation measured by the cell surface stain CFSE and the [^3^H]thymidine incorporation assay. The cell division index (average number of cell divisions) showed a better correlation than percentage of divided cells, since it may determine more accurately the number of cell divisions occurring during culture [17].

Furthermore, using flow cytometry it was possible to quantify spontaneous proliferation in the first 24 hours of culture, compared with [^3^H]thymidine incorporation assay, which requires three to four days of culture. The intensity of proliferation remained stable over a culture period of 120 hours. Sixty-two percent of the individuals infected with HTLV-1 had spontaneous proliferation of PBMCs. This proportion was similar to that found by other authors using the [^3^H]thymidine incorporation assay, which described that 50% to 67% of individuals infected with HTLV-1 has spontaneous proliferation of PBMC [6, 8]. The spontaneous proliferation is one of the most important immunological characteristics of the HTLV-1 infection.

Moreover, the proliferation of CD4^+^ and CD8^+^ T-lymphocyte subsets was quantified. Both CD4^+^ and CD8^+^ T-lymphocyte subsets of infected individuals spontaneously proliferate in culture, without any addition of exogenous antigens or supplementary cytokines, such as IL-2. This proliferation is also observed *in vivo*, and the proliferation rate of the memory CD4^+^ T cells (CD45RO^+^) has been estimated in around 3% per day [7].

Compared with [^3^H]thymidine incorporation assay, classically used to evaluate proliferation in the context of the HTLV-1 infection, the assay using cell staining with CFSE provided a greater number of parameters for statistical analysis is less laborious and expensive process and safer, since it does not require the use of radioactive substances [20–24]. This might be useful to study the pathogenesis of HTLV-1-associated diseases or to evaluate the capacity of drugs to inhibit this process of spontaneous cell proliferation of PBMC from HTLV-1-infected individuals.

## 5. Conclusions

The spontaneous proliferation of PBMC is assessable using flow cytometry. This method may prove useful to better understand the phenomenon of spontaneous proliferation of PBMC of patients infected with HTLV-1.

##  Conflict of Interests

The authors declare that there is no conflict of interests.

## Figures and Tables

**Table 1:** Kinetics of spontaneous proliferation of PBMC from HTLV-1-infected individuals using flow cytometry at different times (*n* = 06).

Measure of	Time (hours)				
proliferation	24	48	72	96	120
Index division*	0.06 ± 0.06	0.05 ± 0.05	0.05 ± 0.06	0.05 ± 0.05	0.06 ± 0.05
% divided cells**	5.1 ± 5.0	4.5 ± 4.8	4.2 ± 5.4	4.0 ± 4.2	4.3 ± 4.0

Median ± Standard Derivation, Kruskal-Wallis test, *P = 0.9; **P = 1.0.
